# Relationships between female infertility and female genital infections and pelvic inflammatory disease: a population-based nested controlled study

**DOI:** 10.6061/clinics/2018/e364

**Published:** 2018-08-06

**Authors:** Xin Tao, Shu-qi Ge, Lei Chen, Li-si Cai, Muh-fa Hwang, Chiung-lang Wang

**Affiliations:** I Center for Reproductive Medicine, the Third Affiliated Hospital, Sun Yat-sen University, Guangzhou, People's Republic of China, 510630; II Department of Infertility and Sexual Medicine, the Third Affiliated Hospital, Sun Yat-sen University, Guangzhou, People's Republic of China, 510630; III Department of Obstetrics and Gynecology, Min-Sheng General Hospital, Taoyuan, Taiwan

**Keywords:** Bacterial Vaginosis, Endometritis, Genital Tract Infection, Infertility, Intrauterine Device, Pelvic Inflammatory Disease

## Abstract

**OBJECTIVES:**

Our purpose was to examine the associations of female genital infections and certain comorbidities with infertility.

**METHODS:**

The Taiwan National Health Research Database was searched for women with a new diagnosis of infertility between 2000 and 2013. Women without a diagnosis of infertility served as a control group and were matched with the infertility cases by age (±3 years) and index year. They were divided into two groups: ≤40 years old and >40 years old. Univariate and multivariate conditional logistic regression models were employed to identify the risk factors associated with infertility.

**RESULTS:**

A total of 18,276 women with a new diagnosis of infertility and 73,104 matched controls (mean cohort age, 31±6.2 years) were included. According to the adjusted multivariate analysis, pelvic inflammatory disease involving the ovary, fallopian tube, pelvic cellular tissue, peritoneum (odds ratio (OR)=4.823), and uterus (OR=3.050) and cervical, vaginal, and vulvar inflammation (OR=7.788) were associated with an increased risk of infertility in women aged ≤40 years. In women aged >40 years, pelvic inflammatory disease of the ovary, fallopian tube, pelvic cellular tissue, and peritoneum (OR=6.028) and cervical, vaginal, and vulvar inflammation (OR=6.648) were associated with infertility. Obesity, lipid metabolism disorders, dysthyroidism, abortion (spontaneous or induced), bacterial vaginosis, endometritis, and tubo-ovarian abscess were associated with an increased risk of infertility according to the univariate analysis but not the multivariate analysis.

**CONCLUSIONS:**

Female genital tract infections, but not the comorbidities studied here, are associated with an increased risk of infertility.

## INTRODUCTION

Female genital infections and pelvic inflammatory disease have been reported to be possible causes of female infertility 1,2. Pelvic inflammatory disease involves infection and inflammation of the upper genital tract (endometrium, fallopian tubes, ovaries, and pelvic peritoneum), and tissue damage caused by infection and inflammation can result in infertility, ectopic pregnancy, and chronic pelvic pain 1-3. Infection spreads from the vagina to the cervix and subsequently to the upper genital tract, with the two most commonly implicated pathogens being *Chlamydia trachomatis* and *Neisseria gonorrhea* 3-6. Pelvic inflammatory disease ranges from a mild or even subclinical disease 7 to an acute severe illness with the development of tubo-ovarian abscesses 8. Endometritis, including subclinical or chronic endometritis, commonly develops in women with conditions such as repeated implantation failure and recurrent miscarriage 4,9. Furthermore, the prevalence of lower genital tract infections, such as bacterial vaginosis, is higher in women with infertility 10-14.

Conditions other than genital tract infections, such as obesity, lipid metabolism disorders, and a history of abortion, have also been suggested to be associated with an increased risk of female infertility 15-19. Despite decades of research, however, the relationships between many conditions and infertility remain unclear.

Thus, the purpose of this study was to use a population-wide database of approximately 23 million people to examine the relationships between female infertility and various female genital tract infections and several comorbidities. The use of a very large dataset may uncover relationships that are not apparent in studies examining fewer patients. Understanding the conditions associated with infertility may aid in the diagnosis and treatment of unexplained infertility.

## MATERIALS AND METHODS

### Data Source and Patients

In 1995, Taiwan instituted the National Health Insurance single-payer program that, by 2009, covered 99% of the population of Taiwan. In 2014, after the initiation of the National Health Insurance program, the National Health Research Institute Database (NHRID) was created and released for research purposes. The NHIRD contains original claims data for more than 23 million people, 99.9% of the entire population of Taiwan, and thus it represents a large population-based resource for epidemiological studies 20,21. The NHRID includes all information on outpatient and inpatient claims data, and all patient information is de-identified. All clinical diagnoses and procedures are recorded according to the International Classification of Diseases, Ninth Revision, Clinical Modification (ICD-9-CM) coding scheme. Detailed information is available on the database website: https://nhird.nhri.org.tw/en/.

### Study Design

This study used a nested case-controlled design with a 1:4 density sampling procedure 20,22. The NHIRD was searched for women with a new diagnosis of infertility made between 2000 and 2013. Infertility was defined as an ICD-9-CM code diagnosis (ICD9=628) (case group). Patients who were first diagnosed with infertility between January 1, 2001, and December 31, 2013, were defined as newly diagnosed infertility cases. Women in the dataset with infertility who had received any previous infertility diagnosis between January 1, 2000 and December 31, 2000 were excluded. The date of the first infertility claim was defined as the index date. Women without a diagnosis of infertility at the index date, who were ever pregnant, and never used any gonadotropins or ovulation stimulants were randomly selected for inclusion in the control group. The controls were individually matched with the infertility cases by age (±3 years) and by index year.

The exclusion criteria were as follows: patients with history of a hysterectomy (ICD9=68.9; OP_CODE=683-687, 689), bilateral ovariectomy (ICD9=65.5, 65.6), cancer (ICD9=140-239), previous chemotherapy (CURE_ITEM_NO1=D2) or radiotherapy (CURE_ITEM_NO1=D1), polycystic ovary syndrome (PCOS: ICD9=256.4), ovarian failure (ICD9=256.3), endometriosis (ICD9=617), adenomyosis (ICD9=617.0), amenorrhea (ICD9=626.0) or Turner syndrome (ICD=758.6).

### Outcome Measures

The primary outcome was the presence of infertility. The independent variables that were examined for their association with infertility were: bacterial vaginosis (ICD9=616.1), endometritis (ICD9=615.9), tubo-ovarian abscess (614.2), pelvic inflammatory disease (ICD9=614-616), obesity (ICD9=278), disorders of lipid metabolism (ICD9=272), and abortion (spontaneous abortion, legally/illegally induced abortion, and unspecified abortion [ICD9=634-638]).

### Statistical Analysis

For the analysis, women were divided into two groups: those ≤40 years old and those >40 years old.

Descriptive statistics of the patients with infertility and the controls were reported as counts and the corresponding percentages. Univariate and multivariate conditional logistic regression models were employed to identify the risk factors associated with infertility. Variables with a value of *p*<0.05 in the univariate analysis were selected and evaluated using multivariate models. All statistical assessments were two-sided and evaluated at the 0.05 level of significance. Statistical analyses were performed using the statistical software package SPSS complex sample module, version 22.0 (IBM Corp, Armonk, NY).

## RESULTS

**Figure 1:**
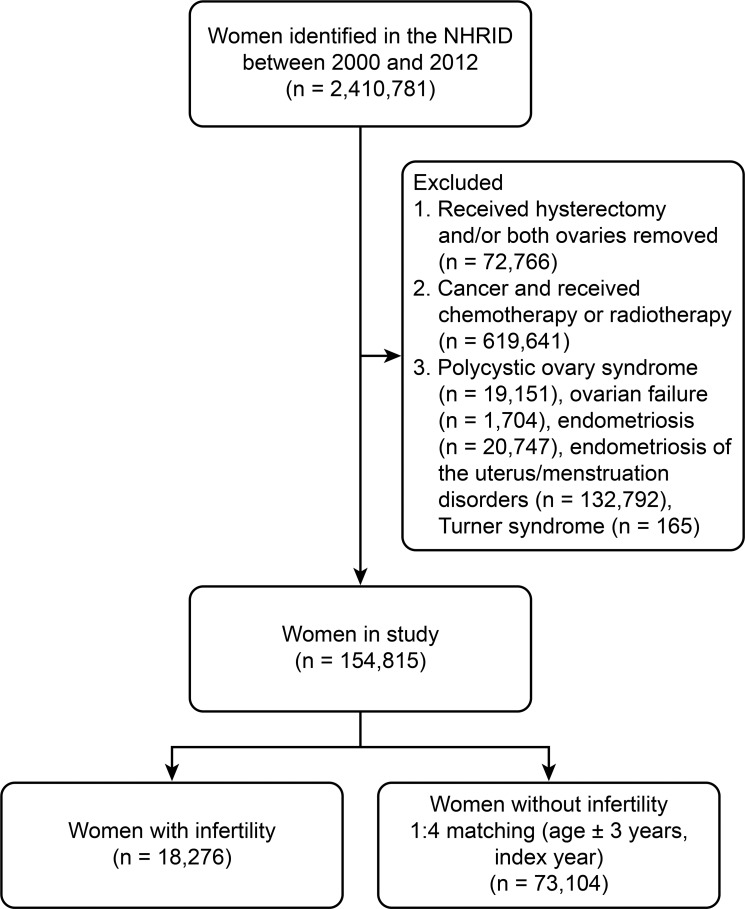
Flow diagram of the patient selection process.

A flow diagram of the patient inclusion process is shown in Figure 1. A total of 2,410,781 women were identified in the NHRID for the period from 2000 to 2012. After applying the exclusion criteria, the analysis included 18,276 women with a new diagnosis of infertility and 73,104 matched controls. The mean age of the study cohort was 31±6.2 years. Patient comorbidities are summarized in Table 1.

**Figure 2:**
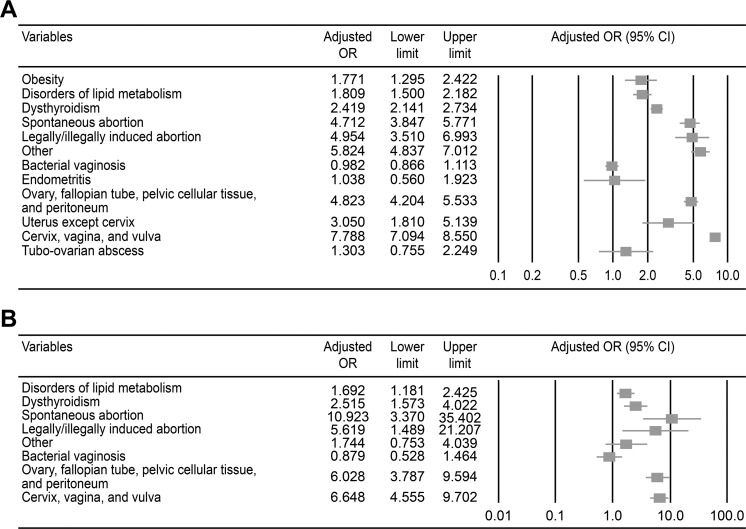
The results of the multivariate logistic regression analysis of risk factors associated with infertility. A) Women ≤40 years old. B) Women >40 years old.

According to the results of the univariate conditional logistic regression analysis, obesity, lipid metabolism disorders, dysthyroidism, abortion (including spontaneous abortion, legally/illegally induced abortion, and other types), bacterial vaginosis, endometritis, pelvic inflammatory disease, and tubo-ovarian abscess were associated with an increased risk of infertility in women aged ≤40 years (all *p*<0.05). After adjusting for obesity, lipid metabolism disorders, dysthyroidism, spontaneous abortion, legally/illegally induced abortion and other types of abortion, the following conditions were significantly associated with an increased risk of infertility in women aged ≤40 years: pelvic inflammatory disease involving the ovary, fallopian tube, pelvic cellular tissue, peritoneum (yes *vs*. no: odds ratio [OR]=4.823, 95% confidence interval (CI): 4.204-5.532), and uterus (yes *vs*. no: OR=3.050, 95% CI: 1.810-5.139) and cervical, vaginal, and vulvar inflammation (yes *vs*. no: OR=7.788, 95% CI: 7.074-8.550) (Table 2 and Figure 2A).

The results of the univariate and multivariate conditional logistic regression models designed to determine the risk factors associated with infertility in women aged >40 years are shown in Table 3. Based on the results from the univariate analysis, lipid metabolism disorders, dysthyroidism, abortion (spontaneous abortion, legally/illegally induced abortion and other types), bacterial vaginosis, endometritis, and pelvic inflammatory disease were associated with infertility (all *p*<0.05). After controlling for confounding factors, the multivariate conditional logistic regression analysis revealed significant associations between pelvic inflammatory disease of the ovary, fallopian tube, pelvic cellular tissue, and peritoneum (yes *vs*. no: OR=6.028, 95% CI: 3.786-9.598) and cervical, vaginal, and vulvar inflammation (yes *vs*. no: OR=6.648, 95% CI: 4.555-9.705) with an increased risk of infertility in women aged >40 years (Figure 2B).

## DISCUSSION

This nested case-controlled study utilizing a nationwide population-based database sought to clarify the factors associated with an increased risk of female infertility. A multivariate analysis adjusted for potential confounders revealed relationships between pelvic inflammatory disease involving the ovary, fallopian tube, pelvic cellular tissue, peritoneum (OR=4.823), and uterus (OR=3.050) and cervical, vaginal, and vulvar inflammation (OR=7.788) with an increased risk of infertility in women aged ≤40 years. Moreover, pelvic inflammatory disease of the ovary, fallopian tube, pelvic cellular tissue, and peritoneum (OR=6.028) and cervical, vaginal, and vulvar inflammation (OR=6.648) were associated with an increased risk of infertility in women aged >40 years.

Female upper genital tract infections and pelvic inflammatory diseases have been reported to result in infertility 1-9,23. The two most common causes of upper genital tract infections in women are *Chlamydia trachomatis* and *Neisseria gonorrhea*, which cause tubal inflammation, damage, and scarring (tubal factor infertility). Other organisms, such as *Mycoplasma genitalium*, *Ureaplasma urealyticum*, and *Trichomonas vaginalis*, may also be involved in the pathophysiology of infertility 24,25. Interestingly, anti-*Chlamydia trachomatis* seropositivity for IgG3 was recently shown to be associated with a lower likelihood of pregnancy, even in the presence of patent fallopian tubes 26. On the other hand, an epidemiological study based on a statistical model found that at the population level, the likelihood of all-cause tubal factor infertility in women with a past or current chlamydia infection was low (0.9% in women aged 25-29 years and 1.4% in women aged 35-39 years); these estimates varied slightly depending on the definition of infertility used 27.

Bacterial vaginosis is a condition in which the normal vaginal lactobacilli flora are replaced by an overgrowth of other microorganisms, including *Gardnerella vaginalis*, anaerobic rods, *Peptostreptococcus* species, and various mycoplasma species 10. The condition has been associated with infertility in a number of studies 12; however, researchers have not yet determined whether bacterial vaginosis is a cause of female infertility or is simply associated with infertility 11. A recent systematic review and meta-analysis reported a significantly higher prevalence of bacterial vaginosis in women with infertility than in fertile women (OR=3.32) and a significantly higher prevalence in women with tubal infertility than in women with infertility caused by other factors (OR=2.77), but bacterial vaginosis was not associated with decreased conception rates (OR=1.03) 12. The authors cautioned that the quality of the included studies was limited and that all studies employed a cross-sectional design that did not allow an inference of causation. However, the authors believed that strong circumstantial evidence supported the hypothesis that bacterial vaginosis infections lead to infertility. Another review conducted in the same year examined whether bacterial vaginosis was associated with pelvic inflammatory disease 11. Based on 17 studies, the authors concluded that little evidence supported a relationship between bacterial vaginosis-associated microorganisms and pelvic inflammatory disease, and no evidence of a causal relationship was observed between bacterial vaginosis and pelvic inflammatory disease.

Abortion, either a single spontaneous abortion or an induced abortion (medical or surgical), is generally not considered a risk factor for infertility. Induced abortion, primarily surgical abortion, has a very low risk of resulting in endometritis, a more serious pelvic infection, or scarring of the uterus (Asherman syndrome), and thus may be associated with infertility for the same reasons as pelvic inflammatory disease. A national cohort study conducted in Scotland found that induced abortion in a woman's first pregnancy increased the risk of spontaneous preterm birth compared with that associated with induced abortion in primigravida women (adjusted risk ratio (aRR)=1.37, 95% CI: 1.32-1.42) or that in women with an initial live birth (aRR=1.66, 95% CI: 1.58-1.74) but not compared with that in women who had a previous miscarriage (aRR=0.85, 95% CI: 0.79-0.91) 19. Compared with medical abortion, surgical abortion increased the risk of spontaneous preterm birth (aRR=1.25, 95% CI: 1.07-1.45). The risk of a spontaneous preterm delivery was not increased after two, three, or four consecutive induced abortions.

Notably, in the current study, patients were stratified into groups by an age greater than or less than 40 because this age is when fertility begins to markedly decrease. The relationship between pelvic infection and infertility was not affected by age, suggesting the possibility that infection and advanced age may exert an additive effect on infertility.

This study has both strengths and limitations. The NHIRD includes medical claims data from 99% of the 23.74 million people in Taiwan, 98% of whom are Han Chinese, and thus represents an excellent data source for population-level analyses and minimizes discrepancies and biases. However, the database does not contain related information, such as personal exposure and lifestyle data, family history, and personal clinical laboratory data. Because infertility treatments are not covered by the NHI, we were not able to evaluate the relationships between factors associated with infertility and specific treatments. We did not examine specific pathogens causing genital tract infections, including cases of pelvic inflammatory disease. Finally, the data were mainly obtained from Chinese Han patients living in Taiwan, and this limitation, along with cultural habits, may not allow the results to be directly applicable to other populations.

The results of this nationwide population-based study confirmed strong relationships between some female genital tract infections and infertility. Importantly, bacterial vaginosis and endometritis were not associated with female infertility.

## AUTHOR CONTRIBUTIONS

Tao X is the guarantor of integrity of the entire study and was responsible for the study conception and design, data analysis and interpretation, critical revision of the manuscript, approval of the final manuscript version. Ge SQ was responsible for the study conception and design, data acquisition, manuscript drafting, approval of the final version of the manuscript and statistical analysis. Chen L was responsible for the data acquisition, data analysis and interpretation, critical revision of the manuscript, approval of the final version of the manuscript and literature research. Cai LS was responsible for the data acquisition, data analysis and interpretation, critical revision of the manuscript, approval of the final version of the manuscript and clinical studies. Hwang MF was responsible for the data acquisition, critical revision of the manuscript and approval of the final version of the manuscript. Wang CL was responsible for the data acquisition, critical revision of the manuscript and approval of the final version of the manuscript.

## Figures and Tables

**Table 1:** Demographic characteristics and comorbidities of the entire cohort. (n = 91,380)

Age (years)	31.1 ± 6.2
**Comorbidities**	
Obesity	217 (0.6)
Lipid metabolism disorders	735 (0.8)
Dysthyroidism	1355 (1.5)
Abortion	
Spontaneous abortion	520 (0.6)
Legally/illegally induced abortion	181 (0.2)
Other	600 (0.7)

Age is reported as the mean ± standard deviation; other data are reported as numbers (percentages).

**Table 2:** Risk factors for infertility in women aged ≤40 years.

	With Infertility(n = 17,315)	Without Infertility(n = 69,260)	Crude OR (95% CI)	Adjusted OR (95% CI)
**Comorbidities**				
Obesity	76 (0.4)	126 (0.2)	**2.419 (1.819-3.217)**	**1.771(1.295-2.422)**
Lipid metabolism disorders	208 (1.2)	364 (0.5)	**2.301 (1.939-2.731)**	**1.809 (1.500-2.183)**
Dysthyroidism	527 (3.0)	746 (1.1)	**2.883 (2.576-3.227)**	**2.419 (2.140-2.733)**
Abortion				
Spontaneous abortion	326 (1.9)	176 (0.3)	**7.532 (6.266-9.055)**	**4.712 (3.847-5.771)**
Legally/illegally induced abortion	111 (0.6)	60 (0.1)	**7.441 (5.433-10.191)**	**4.954 (3.510-6.993)**
Other	373 (2.2)	198 (0.3)	**7.679 (6.458-9.131)**	**5.824 (4.837-7.012)**
**Genital infections**				
Bacterial vaginosis	1655 (9.6)	818 (1.2)	**8.843 (8.117-9.632)**	0.982 (0.866-1.113)
Endometritis	143 (0.8)	70 (0.1)	**8.231 (6.181-10.961)**	1.038 (0.560-1.921)
**Pelvic inflammatory disease, location**				
Ovary, fallopian tube, pelvic cellular tissue, and peritoneum	963 (5.6)	390 (0.6)	**10.400 (9.234-11.712)**	**4.823 (4.204-5.532)**
Uterus, except cervix	204 (1.2)	97 (0.1)	**8.501 (6.672-10.832)**	**3.050 (1.810-5.139)**
Cervix, vagina, and vulva	3159 (18.2)	1554 (2.2)	**9.723 (9.126-10.359)**	**7.788 (7.094-8.550)**
**Tubo-ovarian abscess**	67 (0.4)	22 (0.0)	**12.225 (7.550-19.794)**	1.303 (0.755-2.249)

CI, confidence interval; OR, odds ratio.

Data are reported as numbers (percentages).

Significant values are presented in bold (*p*<0.05).

**Table 3:** Risk factors for infertility in women aged > 40 years.

	With Infertility(n = 961)	Without Infertility(n = 3,844)	Crude OR (95% CI)	Adjusted OR (95% CI)
**Comorbidities**				
Obesity	5 (0.5)	10 (0.3)	2.005 (0.684-5.880)	
Lipid metabolism disorders	51 (5.3)	112 (2.9)	**1.867 (1.331-2.621)**	**1.692 (1.181-2.425)**
Dysthyroidism	32 (3.3)	50 (1.3)	**2.614 (1.667-4.097)**	**2.515 (1.573-4.022)**
Abortion				
Spontaneous abortion	14 (1.5)	4 (0.1)	**14.192 (4.661-43.214**	**10.923 (3.370-35.399)**
Legally/illegally induced abortion	6 (0.6)	4 (0.1)	**6.031 (1.699-21.416)**	**5.619 (1.489-21.209)**
Other	11 (1.1)	18 (0.5)	**2.461 (1.159-5.228)**	1.744(0.753-4.039)
**Genital infections**				
Bacterial vaginosis	82 (8.5)	56 (1.5)	**6.310 (4.457-8.933)**	0.879 (0.528-1.464)
Endometritis	5 (0.5)	4 (0.1)	**5.021 (1.346-18.733)**	—
**Pelvic inflammatory disease, location**				
Ovary, fallopian tube, pelvic cellular tissue, and peritoneum	69 (7.2)	31 (0.8)	**9.515 (6.189-14.626)**	**6.028(3.786-9.598)**
Uterus, except cervix	9 (0.9)	4 (0.1)	**9.076 (2.789-29.533)**	—
Cervix, vagina, and vulva	166 (17.3)	104 (2.7)	**7.509 (5.808-9.707)**	**6.648 (4.555-9.702)**
**Tubo-ovarian abscess**	0	3 (0.1)	—	

CI, confidence interval; OR, odds ratio.

Data are reported as numbers (percentages).

Significant values are shown in bold (*p*<0.05).

— Not included in the model because of the small number of events.
